# Herz-Kreislauf-Erkrankungen und COVID-19: Pathophysiologie, Komplikationen und Therapien

**DOI:** 10.1007/s00059-020-05013-y

**Published:** 2021-01-04

**Authors:** Elisabeth Schieffer, Bernhard Schieffer, Denise Hilfiker-Kleiner

**Affiliations:** 1 Institut für Sportmedizin, Medizinische Hochschule Hannover, Hannover, Deutschland; 2 Klinik für Kardiologie, Angiologie and Intensivmedizin, Philips-Universität Marburg, Marburg, Deutschland; 3 Klinik für Kardiologie und Angiologie, Medizinische Hochschule Hannover, Carl-Neuberg-Str. 1, 30625 Hannover, Deutschland

**Keywords:** Coronavirus, Pandemie, Herz-Kreislauf-System, Behandlung, Prävention, Coronavirus, Pandemic, Cardiovascular system, Treatment, Prevention

## Abstract

COVID-19 („coronavirus disease 2019“) ist eine Herausforderung für unser Gesundheitssystem und gleichzeitig eine der herausragenden Katalysatoren erfolgreicher translationaler Forschung. COVID-19 ist nicht nur eine simple Viruserkrankung des Bronchialsystems, sondern eine pandemisch auftretende, hyperinflammatorische Multiorganerkrankung. Das Herz-Kreislauf-System spielt dabei eine kausale Rolle, da SARS-CoV‑2 („severe acute respiratory syndrome coronavirus 2“) Wirtszellen über ACE(„angiotensin-converting enzyme“)-2, ein Enzym des Renin-Angiotensin-Systems, befällt. Darüber hinaus spielen kardiovaskuläre Komorbiditäten und Risikofaktoren wie Bluthochdruck, Diabetes und Adipositas eine wichtige Rolle für die Schwere der Krankheitsverläufe. Zusätzliche Risikofaktoren wie Geschlecht, Alter, Genetik und Luftverschmutzung modulieren sowohl die Schwere der SARS-CoV-2-Infektion als auch kardiovaskuläre Erkrankungen. Als Folge von COVID-19 kommt es zu vermehrten Thrombosen, Herzinfarkten, Herzmuskelentzündungen und Vaskulitiden, die das kardiovaskuläre System direkt schädigen und wesentlich zur Morbidität und Mortalität beitragen. Erkenntnisse aus zahlreichen Studien zu Krankheitsverläufen von SARS-CoV-2-infizierten Patienten haben zu besseren Therapiemöglichkeiten geführt, die nun in der zweiten Welle zum Teil standardisiert und insbesondere auch an Komplikationen des kardiovaskulären Systems angepasst wurden und werden. In diesem Review geben wir einen kurzen Überblick über die Pathophysiologie des SARS-CoV-2-Virus allgemein sowie auch spezifisch auf das kardiovaskuläre System. Daraus folgend, fassen wir die aktuellen Therapieansätze und deren pathophysiologische Grundlagen (Stand November 2020) zusammen.

## Abstract (en)

The coronavirus disease 2019 (COVID-19) pandemic is a challenge for our healthcare system but at the same time is one of the excellent catalyzers and promoters of successful translational research. The COVID-19 is not only a simple viral infection of the bronchial system but is also a pandemic hyperinflammatory multiorgan disease. The cardiovascular system plays a causal role in this context, as severe acute respiratory syndrome coronavirus 2 (SARS-CoV-2) invades host cells via the angiotensin-converting enzyme 2 (ACE-2), an enzyme in the renin-angiotensin system. Furthermore, cardiovascular comorbidities and risk factors, such as hypertension, diabetes and obesity play an important role in the severity of the course of the disease. Additional risk factors, such as gender, age, genetics and air pollution modulate both the severity of the SARS-CoV‑2 infection as well as cardiovascular diseases. As sequelae of COVID-19, increased thrombosis, myocardial infarction, myocardial inflammation and vasculitis occur, which directly damage the cardiovascular system and substantially contribute to the high morbidity and mortality. Knowledge gained from many studies on the course of the disease in patients infected with SARS-CoV‑2 has led to improved treatment possibilities, which now in the second wave are partly standardized and were, and are, in particular adapted to complications of the cardiovascular system. In this review we provide a short overview on the pathophysiology of the SARS-CoV‑2 in general and also specifically on the cardiovascular system. Furthermore, we summarize the current treatment approaches and their pathophysiological principles (status November 2020).

## Einleitung

Die COVID-19(„coronavirus disease 2019“)-Pandemie hält die Welt seit Ende 2019 in Atem. Lief die erste „Welle“ der Erkrankung in Deutschland noch vergleichsweise glimpflich ab, trifft uns die zweite Welle im Herbst 2020 mit deutlich höheren Infektions‑, Erkrankungs- und Mortalitätszahlen. Unser Gesundheitssystem arbeitet in manchen Regionen am Rande der Dekompensation, infizierte Mitarbeiter und Kollegen sind ebenso wie schwerstkranke Patienten an der extrakorporalen Membranoxygenierung zu unserem Alltag geworden. Die rasche Verbreitung des neuen Pathogens zeigte erstmals Nachteile des globalen Handels- und Reiseverkehrs auf, und in Europa sind Namen wie Ischgl, Heinsberg und Bergamo untrennbar mit der COVID-19-Pandemie verbunden. Ironischerweise wurden Hotspots nicht zum Sinnbild der Digitalisierung Deutschlands, sondern zum Synonym der COVID-19-Pandemie.

Aber die durch SARS-CoV2 („severe acute respiratory syndrome coronavirus 2“) verursachte Pandemie hat einen niemals dagewesenen Schulterschluss von Wissenschaftlern und Klinikern initiiert und damit einen Erkenntnisgewinn ausgelöst, der schlussendlich in einem translationalen Brückenschlag zur raschen Impfstoffentwicklung und weltweit differenzierten und standardisierten Therapie von COVID-19-Erkrankten geführt hat.

**Figure 1:**
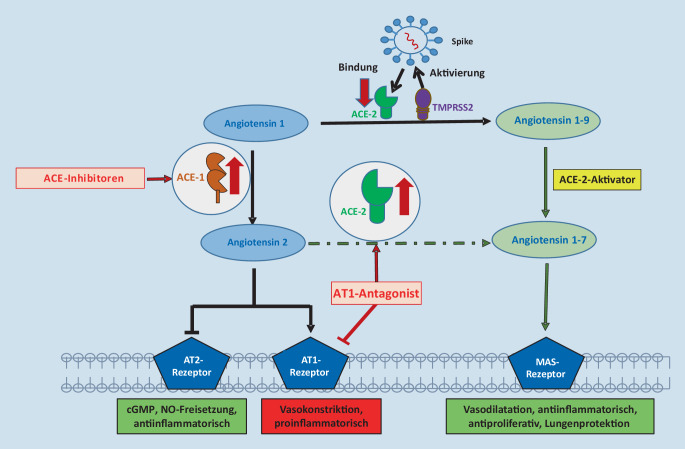

Auf dem Boden der Daten der SARS-CoV-1-Epidemie in China (2002–2004) war bisher bekannt, dass das Virus über ACE(„angiotensin-converting enzyme“)-2, ein Schlüsselenzym in der Blutdruckregulation, seine Wirtszellen befällt (Abb. 1). Nach anfänglichen Bedenken bezüglich potenziell negativer Effekte antihypertensiver Therapien mit ACE-Inhibitoren (ACE-I) oder AT1(Angiotensin-II-Rezeptor Subtyp 1)-Antagonisten (Angiotensinrezeptorblocker, ARB) zeigen nun mehrere Studien, dass beide Medikamentengruppen vermutlich positive Effekte bei COVID-19-Patienten haben (Abb. 1; [1, 2]).

Schon früh hat sich gezeigt, dass COVID-19 nicht nur die Atemwege betrifft, sondern dass das Herz-Kreislauf-System genauso betroffen ist. So deutete die auffällige Häufung von Thrombosen, Myokardinfarkten und Schlaganfällen auf Störungen des Gerinnungssystems und eine Gefäßbeteiligung hin. Ebenfalls scheint eine generelle Störung des Immunsystems vermehrt zu Myokarditiden und Vaskulitiden zu führen [3, 4]. Neuere Evidenzen implizieren, dass es über immunthrombotische Mechanismen Verbindungen zwischen dem Immunsystem, COVID-19 und kardiovaskulären Komplikationen gibt. Kardiovaskuläre Komorbiditäten, insbesondere Bluthochdruck, Adipositas und Diabetes, sind zudem Risikofaktoren für schwere COVID-19-Verläufe. Zusätzliche Risikofaktoren wie Geschlecht, Alter, Genetik und Luftverschmutzung modulieren sowohl die Schwere der SARS-CoV-2-Infektion wie auch kardiovaskuläre Erkrankungen. Basierend auf diesen pathophysiologischen Erkenntnissen sowie zahlreichen Studien zu Krankheitsverläufen von SARS-CoV2-infizierten Patienten, sind erste vielversprechende Therapien für COVID-19-Patienten entwickelt worden, wie z. B. eine frühzeitige Antikoagulation und entzündungshemmende Therapie in der Frühphase mit Dexamethason [5]. Auch das Management von kardiovaskulären Patienten mit COVID-19 ist verfeinert worden, sodass nun deutlich bessere Therapiemöglichkeiten für die Bewältigung der zweiten Welle zur Verfügung stehen. In diesem Review fassen wir die Historie von SARS-CoV‑2 zusammen, geben einen Überblick über die Pathophysiologie von COVID-19 allgemein und spezifisch für das kardiovaskuläre System und stellen kurz die aktuellen Therapieansätze und deren pathophysiologische Grundlagen (Stand November 2020) vor.

## Kardiovaskuläre Pathophysiologie bei COVID-19

### Historie und Infektionspathologie von SARS-COV-2

Ein aktueller Überblick über die Virushistorie und die Virusfunktionalität sowie die Pathologie sind im Folgenden zusammengefasst [6, 7]. SARS-CoV‑2 ist das siebte bekannte Coronavirus mit humanpathogener Relevanz. 3 Coronaviren, nämlich SARS-CoV, MERS („Middle East respiratory syndrome“) und SARS-CoV‑2, führen zu epidemischen/pandemischen Verläufen mit schweren Krankheitssymptomen, wohingegen 4 Virusstämme eher milde Krankheitsverläufe zeigten. SARS-CoV‑2 ist, wie auch SARS-CoV und MERS, ein zoonotisches Virus, welches vom Tier auf den Menschen überspringen konnte. Genetische Analysen deuten darauf hin, dass die chinesische Hufeisennasenfledermaus das natürliche Reservoir für SARS-CoV‑2 ist. Andere Säugetiere, wie die im Oktober 2020 entdeckte mutierte Form in Zuchthermelinen in Dänemark, scheinen ebenfalls Wirtstiere von SARS-CoV‑2 zu sein und führen über Spontanmutationen von SARS-CoV‑2 zu unabsehbaren Folgen für den Menschen. SARS-CoV und SARS-CoV‑2 sind RNA-Viren, bestehend aus Einzelstrang(„single-stranded“)-RNA (ssRNA), und haben auf genetischer Ebene eine 80 %ige Ähnlichkeit. Beide Viren befallen humane Zellen über den ACE-2-Rezeptor, dessen Expression nicht nur auf die Atemwege und die Lunge beschränkt ist, sondern von verschiedenen Zellen in den meisten Organen exprimiert wird. Es ist deshalb nicht erstaunlich, dass SARS-CoV‑2 in fast allen Geweben nachgewiesen werden kann, in hohen Konzentrationen v. a. in Herz, Niere, Darm, Blutgefäßen und Gehirn. Diese Variabilität in der SARS-CoV-2-Expression ist einerseits eine Erklärung für die Symphonie an Symptomen, mit der COVID-19 beginnen kann. Andererseits mag es eine Erklärung für den Erfolg einer replikationshemmenden Therapie mit Remdesivir sein, welches heute integraler Bestandteil der frühen COVID-19-Therapie ist. Ebenfalls wichtig für die Infektion von Zellen durch SARS-CoV‑2 ist TMPRSS2 („transmembrane serine protease subtype 2“), einer Protease, die das E(„envelope“)-lokalisierte trimerische S(„spike“)-Protein des Virus spaltet und dem Virus damit ermöglicht, mit der Wirtszellmembran zu fusionieren, sein Genom zu internalisieren und so seine Vermehrung zu induzieren (Abb. 1). Diese hoch konservierte Struktur unterliegt nach heutigen Erkenntnissen keiner Mutation und wurde erfolgreich als Angriffspunkt für die deutsch-amerikanische Impfstrategie von Biontech/Pfizer eingesetzt, bei der die RNA über Liposomen in körpereigene Zellen geschleust wird, sodass sich virusspezifische Antikörper über einen langen Zeitraum bilden können.

### Kardiale und vaskuläre Pathologien bei COVID-19-Patienten

Endothelzellen exprimieren den ACE-2-Rezeptoren und können somit auch direkt mit SARS-CoV‑2 infiziert werden [8]. Pathologische Herzveränderungen, Verletzungen des vaskulären Endothels und Mikroangiopathien sind bei COVID-19-Patienten eine häufige Komplikation [9, 10]. Myokardschädigungen werden bei etwa 20 % der COVID-19-Patienten beobachtet und sind mit einem erhöhten Mortalitätsrisiko verbunden [3, 11, 12]. Auffallend häufig ist dabei v. a. die rechtsventrikuläre (RV) Dilatation (39 %), gefolgt von einer linksventrikulären (LV) diastolischen Dysfunktion (16 %), wie man sie bei einer floriden Virusmyokarditis im Anfangsstadium auch sieht; mit etwa 10 % eher selten sind systolische LV-Funktions-Störungen. Eine routinemäßige echokardiographische Untersuchung von COVID-19-Patienten wird deshalb in kürzeren Abständen je nach Erkrankungsverlauf empfohlen. Bei schweren Verläufen kann ein zusätzliches frühzeitiges Management mit einem Pulmonaliskatheter in den Händen erfahrener Intensivmediziner hilfreich sein. Als Ursache der RV-Dilatation wird eine unkontrollierte Steigerung des Pulmonalisdrucks durch die Virusinfektion postuliert.

Ebenfalls häufig sind ventrikuläre Arrhythmien und hämodynamische Instabilität ohne koronare Herzkrankheit [10, 13, 14]. In post-mortem-Analysen von 22 Patienten, die an COVID-19-bedingtem respiratorischen Versagen gestorben waren, fanden sich massiv dilatierte RV [15, 16], die als Zeichen der RV-Belastung beim akuten Lungenversagen („acute respiratory distress syndrome“, ARDS) zu interpretieren sind. Es wird aber auch vermutet, dass SARS-CoV‑2 über Infektion von perimyozytären Zellen, v. a. von mikrovaskulären Endothelzellen, zu direkten kardialen Schädigungen in Form einer myokardialen Mikrovaskulopathie und über inflammatorische Mediatoren zu einer stressinduzierten Kardiomyopathie führen kann. Verstärkt freigesetzte Herzenzyme [10], hier v. a. von hs(„high sensitive“)-Troponin, Kreatinkinase (CK)/CK-MB („muscle-brain type“) und NT-proBNP („N-terminal pro brain natriuretic peptide“), stellen deshalb routinemäßige Laborparameter dar, die eine echokardiographische und klinische Einschätzung einer SARS-CoV-2-vermittelten Myokardschädigung unterstützen [10, 13]. In der ECHOVID-19-Studie korrelierten reduzierte Werte im LV- und RV-Strain mit einem schweren klinischen Verlauf von COVID-19 [17].

Das vaskuläre System ist aber nicht nur im Myokard, sondern in allen Organen für die systemische Verbreitung von SARS-CoV‑2 verantwortlich, und der ACE-2-Rezeptor ist die Eintrittspforte für das Virus [8]. So zeigten z. B. post-mortem-Untersuchungen an Lungengewebe von an COVID-19 verstorbenen Patienten massive Schädigungen des vaskulären Endothels mit Mikroangiopathien und Thromben, die ein deutlich stärkeres Ausmaß aufweisen, als das bei Influenzainfektionen oder bei anderen schwer kranken Patienten ohne SARS-CoV-2-Infektion beobachtet wird [11, 12]. Möglicherweise spielt dabei eine verstärkte Komplementaktivierung im vaskulären Endothel eine Rolle, die dann zu Endothelitis und Hypoxie in verschiedenen Organen führt [4]. Standardisierte klinische Untersuchungsmethoden zur Erfassung der vaskulären/endothelialen Beteiligung bei COVID-19-Patienten, wie Bioimpedanz oder Kapillarmikroskopie, sind aktuell in der Erprobung.

Eine schwere Entzündung des Blutgefäßsystems könnte auch bei Kindern und Jugendlichen für schwere Verläufe verantwortlich sein. Insbesondere würde sie das gehäufte Auftreten von MIS‑C („multisystem inflammatory syndrome“), eines dem Kawasaki-Syndrom (KS) ähnlichen Syndroms, bei an COVID-19 erkrankten Kindern erklären [18]. Die meisten Kinder mit KS oder MIS-C-Komplikationen erholen sich gut von COVID-19, wobei hier darauf hingewiesen wird, dass KS oder MIS‑C selten in der akuten SARS-CoV-2-Infektion auftreten, sondern meist etwas später diagnostiziert werden. Dies wiederum deutet darauf hin, dass es eher eine Folge der Schädigung des Gefäßbetts und einer deregulierten Immunantwort und nicht eine direkte Reaktion auf die SARS-CoV-2-Infektion ist. Auf diese Hypothese wird im folgenden Kapitel noch näher eingegangen. Weitere Komplikationen bei Kindern mit COVID-19 sind analog zur adulten Verlaufsform von COVID-19 Arrhythmien, reduzierte Herzfunktion und erhöhte Troponinspiegel sowie Koronararterienaneurysmata und Myokardinfarkte [18, 19].

Zusammenfassend deuten alle klinischen Befunde daraufhin, dass der systemischen Endothelitis durch den Befall mit SARS-CoV‑2 ein maßgeblicher Anteil des Multiorganversagens zuzuschreiben ist. Welchen Einfluss ACE-Hemmer, AT1-Blocker und Statine auf diesen Effekt haben, ist unklar. Da diese Substanzen aber die endotheliale Funktion verbessern, sollten sie bei allen COVID-19-Patienten fortgeführt werden.

### Pathologische Veränderungen des Immun- und Gerinnungssystems bei COVID-19

Die Immunthrombose wird als mögliche Ursache für das erhöhte Thromboserisiko bei COVID-19-Patienten verantwortlich gemacht. Die Immunthrombose ist ein Prozess des angeborenen Immunsystems, bei dem Thrombozyten und Gerinnungsfaktoren eingedrungene Pathogene binden, um diese zu neutralisieren. Über Membranbestandteile von Thrombozyten und Komplexen aus Komplement und Thrombozyten kommt es begleitend zu einer erhöhten Zytokinproduktion, insbesondere von Interleukin(IL)-6, IL‑1 und TNF(Tumornekrosefaktor)-alpha. Bei SARS-CoV-2-Infektionen könnte die Aktivierung des angeborenen Immunsystems über die Immunthrombosereaktion sowohl für eine verstärkte Entzündungsreaktion wie auch eine erhöhte Gerinnungsaktivität verantwortlich sein [20]. Auch könnte eine Verbindung zum RAAS und insbesondere zu ACE‑2, z. B. über die Kallikrein-Bradykinin-Achse, eine Koagulopathie begünstigen. Welche Rolle hierbei die ACE-1-ähnliche Chymase in perivaskulären monozytären Zellen hat, ist im Moment unklar. Histopathologische Befunde legen aber den Schluss nahe, dass eine verstärkte Aktivierung von neutrophilen Granulozyten und eine Komplementaktivierung Teile der immunothrombotischen Mechanismen bei COVID-19 ausmachen könnten [20].

Die Immunpathologie von COIVD-19 zeigt weiterhin, dass nicht nur das angeborene, sondern auch das adaptive Immunsystem durch SARS-CoV‑2 verändert wird. Für das adaptive Immunsystem wird neben der Hochregulation und Aktivierung der Neutrophilen eine relative Lymphozytopenie beschrieben [7]. Niedrige Lymphozytenpopulationen (CD3+, CD4+ und Cd8+) scheinen mit stärkerer Organverletzung und schwerer Lungenentzündung sowie einer erhöhten Mortalität bei hospitalisierten Patienten korreliert zu sein [21]. Es häufen sich zudem Berichte, dass die SARS-CoV-2-Infektion zu einer Reduktion von residenten wie auch zirkulierenden T‑Zellen („T-cell exhaustion“) führt [22, 23]. Für B‑Zellen wird berichtet, dass schwere COVID-19-Verläufe mit einer deutlichen Reduktion von gewebeständigen und zirkulierenden B‑Zellen assoziiert sind, die sich z. B. in einem Verlust der „germinal centers“ und der „Bcl-6+ germinal center B‑cells“ zeigt [24]. Eine weitere Abnahme des B‑Zell-Pools bei SARS-CoV-2-Infektionen könnte auch aus einer zusätzlichen Transformation von Plasmazellen zu einer neuartigen Neutrophilenpopulation bei SARS-CoV-2-Infektionen resultieren [23]. Weitere Studien zeigten, dass Patienten mit schlechter funktionierenden B‑Zellen aufgrund von genetischen Varianten schwerere Verläufe aufwiesen als solche, die diese Varianten nicht tragen [25], während das Risiko für schwere Verläufe bei Patienten mit größeren Pools von naiven B‑Zellen geringer ist [26].

Zudem gibt es Hinweise darauf, dass über die SARS-CoV-2-Infektion aktivierte Antikörper die Entzündungsreaktion verstärken und evtl. auch die Bildung von Autoantikörpern fördern. Dies könnte dann als Folgereaktion auf COVID-19 zu Zell- und Gewebeschädigungen führen und z. B. das KS oder das MIS‑C bei Kindern auslösen. Für SARS-CoV‑1 wurde bereits gezeigt, dass SARS-CoV-1-Antikörper die Entzündungsreaktion in Primaten und humanen Makrophagen amplifizieren [27]. Auch gibt es Daten, die darauf hindeuten, dass genetisch bedingte Defekte

- in TLR („toll-like receptor 3“), einem Rezeptor der angeborenen Immunität und
- im Typ-1-Interferon(IFN)-Signalling oder
- IFN-Autoantikörper, die IFN neutralisieren,

mit schwereren COVID-19-Verläufen assoziiert sind [28].

Die pathologischen Veränderungen des adaptiven Immunsystems durch SARS-CoV‑2 verhindern auch eine effiziente Bildung von Gedächtnis-T- und B‑Zellen, die für die Ausbildung der Immunität von COVID-19-Patienten nötig wären – eine Problematik, die durch die sich mehrenden Berichte von Reinfektionen verschärft wird [29, 30]. Erfreulicherweise scheint aber doch bei vielen Patienten eine Langzeitimmunität zu bestehen, da Gedächtniszellen gegen COVID-19 bis zu 8 Monate nach Infektionsabheilung nachgewiesen wurden.

Zusammenfassend ergibt sich ein komplexes Bild der Immunreaktion auf eine SARS-CoV-2-Infektion. Diese beinhaltet sowohl eine immunthrombotische Antwort des adaptiven Immunsystems, welches Viren zwar neutralisiert, aber gleichzeitig das Risiko für Thrombosen erhöht, wie auch eine durch SARS-CoV-2-Infektion induzierte Schwächung des adaptiven Immunsystems, die eine Virusvermehrung begünstigt und die Entwicklung einer effizienten Langzeitimmunität verhindert. Als Folge eines so deregulierten Immunsystems erhöht sich das Risiko für Sekundärinfektionen mit Bakterien und Pilzen, wie auch für Reinfektionen durch SARS-CoV‑2. Zudem kann es zu Sekundäreffekten wie Autoimmunreaktionen und massiven Entzündungen, vermutlich nach der Akutphase der SARS-CoV-2-Infektion, kommen, die im Extremfall einen Zytokinsturm und Multiorganversagen auslösen können [7]. V. a. unsere Impfstrategie stellt die oben beschriebene komplexe Immunpathologie vor eine große Herausforderung, repetitive Impfungen versus „Boosterungen“ wie bei Hepatitis C sind aktuell in der Diskussion. Auch bleibt abzuwarten, ob neue durch RNA-Transduktion induzierte Impfstrategien breit verträglich sind oder evtl. auch Autoimmunreaktionen hervorrufen könnten.

## Risikofaktoren für COVID-19

### Umweltfaktoren

Mit dem ersten Shutdown in Deutschland am 15. März 2020 rückten auch bei uns Umweltfaktoren, die das SARS-CoV-2-Infektions-Geschehen negativ oder positiv beeinflussen würden, in den Fokus. Hohe Temperaturen und Ultraviolett(UV)-Strahlung scheinen die Viruslast zu reduzieren, wohingegen die Viren bei kalten Temperaturen länger überleben. Die Lipidhülle von SARS-CoV‑2 verfestigt sich bei Kälte, und Viren lassen sich bei 4*C noch nach 14 Tagen nachweisen [31]. Diese hohe Stabilität bei Kälte dürfte auch Übertragungswege, z. B. in Schlachthöfen oder Logistikzentren, begünstigen. Luftqualität und Feinstaubbelastung scheinen weitere Faktor zu sein, die die Verbreitung von SARS-CoV‑2 und auch die Krankheitsverläufe von COVID-19-Patienten beeinflussen [32]. Thomas Münzel und sein Team aus Mainz berichten in dieser Ausgabe von *Herz* detailliert über diese Faktoren. Von zentralem Interesse sind hierbei die immunpathogenen Effekte dieser Umwelteinflüsse, die erstmals im Februar 2020 für die hohe Mortalitätsrate in der Region Lombardei in Norditalien verantwortlich gemacht wurden.

### Einfluss von Ethnien und sozialen Faktoren

Afrikanische, asiatische oder hispanische Ethnien scheinen ebenfalls ein höheres Risiko für schwere COVID-19-Verläufe aufzuweisen [33, 34]. Inwiefern dies tatsächlich mit dem genetischen Hintergrund verbunden ist und/oder auch von den Lebens- und Umweltbedingungen abhängt, ist aber unklar [33, 34]. In der SARS-CoV-1-Epidemie in China und Ostasien wurde erstmals ein unterschiedlicher Erkrankungsverlauf für Han- und Non-Han-Chinesen (z. B. Vietnamesen) beschrieben. Für Deutschland liegen bisher nur wenige Daten vor. Eine Erhebung der Allgemeinen Ortskrankenkasse (AOK) und des Instituts für Medizinische Soziologie der Uniklinik Düsseldorf zeigte, dass Langzeitarbeitslose ein um 84 % erhöhtes Risiko für einen COVID-19-bedingten stationären Aufenthalt aufweisen (https://www.aok.de/pk/rh/inhalt/covid-19-und-soziale-unterschiede-1/). Faktoren, die zu sozioökonomischen Unterschieden beitragen können, sind beengte Wohnverhältnisse, Arbeitsbedingungen, die einen Schutz vor Ansteckung erschweren können, Sprachbarrieren oder kulturelle Gewohnheiten, Ernährung und Lifestyle. Insbesondere scheinen indigene und arabische Bevölkerungsgruppen evtl. eine höhere Mortalität aufzuweisen. Hierbei müssen aber auch unterschiedliche Umwelt‑, Ernährungs- und Lebensgewohnheiten mitberücksichtigt werden. Sicher scheint aber zu sein, dass u. a. in der Bevölkerungsgruppe der über 250.000 chinesischen Gastarbeiter in Norditalien schon viel früher die Durchseuchung mit SARS-CoV‑2 stattgefunden hat, als dies von den italienischen Behörden entdeckt wurde. Umweltfaktoren, individuelle Risikofaktoren, eine überalterte Bevölkerung sowie ein überlastetes Krankenhaussystem trugen sicherlich entscheidend zu einer kurzfristigen Übersterblichkeit von Februar bis April in der Lombardei bei.

### Einfluss von Geschlecht und Alter

Während das Risiko, sich zu infizieren, für Frauen und Männer annähernd gleich zu sein scheint, ist das Risiko, einen schweren Verlauf zu entwickeln und an der Infektion zu versterben, für Männer, insbesondere ältere Männer, deutlich höher als für Frauen [35–37]. Als mögliche Gründe für die geschlechterspezifischen Unterschiede von COVID-19-Verläufen werden u. a. Unterschiede in der Expression von ACE‑2 und TMPRSS2 vermutet. Obwohl Männer im Vergleich zu Frauen höhere ACE-2-Spiegel aufweisen, ist der Quotient aus ACE-1- und ACE-2-Spiegel aber wegen eines deutlich höheren ACE-1-Spiegels bei Männern der ungünstigere Faktor. So scheinen Komorbiditäten, die zur höheren ACE-2-Expression führen, bei Männern häufiger zu sein [38]. Umso wichtiger ist eine konsequente therapeutische Blockade des RAAS mit ACE-Hemmern bzw. AT1-Antagonisten bei unseren Patienten.

Zum anderen wird die Expression von TMPRSS2 auch über Androgene und insbesondere den Androgenrezeptor kontrolliert [38]. Geschlechtshormone können auch das Immunsystem unterschiedlich beeinflussen; z. B. fördern Östrogene das adaptive Immunsystem und könnten so zu einer effektiveren Bekämpfung der Virusinfektion bei Frauen führen [38]. Weitere mögliche Pathomechanismen, die das Immunsystem betreffen und die abhängig vom biologischen Geschlecht den Verlauf von COVID-19 beeinflussen, sind im Review von Haverfield et al. [39] zusammengefasst.

Der herausragende Faktor für das Infektionsrisiko, schwere Krankheitsverläufe und eine erhöhte COVID-19-Mortalität ist aber das Alter. Berichte aus China und den USA deuten auf eine bis zu 20-fach höhere Wahrscheinlichkeit für schwere Verläufe von über 60-jährigen Patienten im Vergleich zu jüngeren Patienten hin [40]. Als Gründe werden Seneszenz des Immunsystems und die höhere Prävalenz für Komorbiditäten, insbesondere für Lungenerkrankungen wie COPD („chronic obstructive pulmonary disease“), kardiovaskuläre und onkologische Erkrankungen, aufgeführt [41], auf die wir im Folgenden eingehen werden.

### Einfluss von Begleiterkrankungen

Begleiterkrankungen erhöhen das Risiko für schwere COVID-19-Verläufe [36, 37, 42]. Eine Metaanalyse zeigte, dass Adipositas das Risiko für COVID-19 um etwa 46 %, für eine stationäre Aufnahme um 113 % und für die Aufnahme auf Intensivstation um 74 % sowie das Sterberisiko um 48 % steigert [43]. Neben Adipositas und Diabetes mellitus gehören v. a. auch arterieller Hypertonus und kardiovaskuläre Erkrankungen zu Risikofaktoren für schwere COVID-19-Verläufe [42, 43]. Ebenso sind COPD, Nierenerkrankungen sowie zerebrovaskuläre und Tumorerkrankungen mit einer erhöhten Wahrscheinlichkeit von schwer oder fatal verlaufender COVID-19 assoziiert [36, 37].

## Prävention und Therapieempfehlungen für COVID-19

Im Rahmen von präventiven Maßnahmen sind z. B. immunmodulierende und antiinflammatorische Effekte einer gesunden Lebensweise bereits früh im Pandemiegeschehen diskutiert worden [44, 45]. Bisher liegen aber noch keine epidemiologischen Daten oder Interventionsstudien vor, die den Zusammenhang zwischen Ernährung, Bewegungsverhalten und COVID-19 untersuchten. Die vielfältigen förderlichen Effekte eines moderaten körperlichen Ausdauertrainings und einer mediterranen Ernährungsweise auf das Immunsystem und die Endothelfunktion sind hinreichend belegt. Niedrige Vitamin-D-Spiegel sind ebenfalls mit einem höheren Risiko für eine invasive Beatmung und einer erhöhten Mortalität assoziiert [46, 47]. Interventionsstudien zur Supplementierung von Vitamin D bei COVID-19 sind initiiert, Ergebnisse stehen aber noch aus.

Die Akuttherapie von Patienten mit mittelschweren und schweren Verläufen erfolgt im Allgemeinen stationär. Die Therapieregime unterliegen gerade in diesem Bereich einer hohen Dynamik, angepasst an laufende Studienergebnisse und Empfehlungen von Fachgesellschaften:

- Die Gabe von Remdesivir zur Hemmung der Virusreplikation zeigte in einer randomisierten Studie mit 1062 Patienten eine verkürzte Krankheitsdauer um 10 Tage in der Therapiegruppe im Vergleich zu 15 Tagen in der Kontrollgruppe [48]. Remdesivir ist in Europa für die Behandlung von SARS-CoV-2-bedingten Pneumonien mit Sauerstoffbedarf zugelassen.
- Aufgrund der Endothelschädigungen und des mikro- und makrovaskulären Thromboserisikos werden bei stationären Patienten die Gabe von niedermolekularem Heparin und bei Indikation ggf. eine therapeutische Antikoagulation empfohlen (RKI, Stellungnahme COVID-19-Therapie, Stand 05.11.2020).
- Für unspezifische Immunmodulatoren wie Dexamethason konnte im Recovery Trial bei intubierten COVID-19-Patienten eine Reduktion der Mortalität gezeigt werden, sodass die Gabe bei schweren Verläufen empfohlen wird [5]. In der ganz frühen Phase von COVID-19 können nichtsteroidale Antirheumatika (NSAR) wie Ibuprofen kurzfristig eingesetzt werden, diese sollten aber aufgrund der Drucksteigerung im Pulmonaliskreislauf nicht längerfristig gegeben werden. Zur antipyretischen Therapie kann Metamizol oder Paracetamol eingesetzt werden.
- Für blutdrucksenkende Medikamente wie ACE-Hemmer und AT1-Blocker zeigte die Brace-Corona-Studie, eine prospektiv randomisierte Studie mit 659 SARS-CoV-2-Patienten, die bereits ACE-Hemmer oder AT1-Blocker einnahmen, dass es keinen Unterschied zwischen der Gruppe, die ACE-Hemmer oder AT1-Blocker absetzte und derjenigen, die die Einnahme unverändert fortsetzte, gibt. Neuere Metaanalysen deuten sogar auf potenzielle Überlebensvorteile von mit ACE-Hemmern/AT1-Blockern behandelten hypertensiven COVID-19-Patienten hin [1, 2]. Die Deutsche Gesellschaft für Kardiologie hat bereits früh eine Stellungnahme hierzu verfasst und empfohlen, Therapien mit ACE-Hemmern und AT1-Blockern fortzuführen.
- Auch Statine könnten einen positiven Effekt bei COVID-19-Patienten aufweisen, da sie neben lipidsenkenden auch antiinflammatorische Effekte haben. Erste Evidenzen in einer retrospektiven Analyse von 2736 COVID-19-Patienten deuten auf einen Überlebensvorteil von Patienten mit bestehender Statintherapie gegenüber Patienten ohne Statintherapie hin [41].
- Immunmodulatorische Medikamente, wie Inhibitoren inflammatorischer Zytokine (Tocilizumab gegen IL‑6, Anakinra gegen IL-1), Tyrosinkinaseinhibitoren wie Ruxolitinib gegen Januskinasen, aber auch Plasmapheresetherapien befinden sich in unterschiedlichen Testphasen und haben positive Effekte auf das Überleben in kleinen Patientenserien gezeigt [49].
- Bei schwerer COVID-19 wird zusätzlich auch die venovenöse ECMO(„extracorporeal membrane oxygenation“)-Therapie eingesetzt. Die Daten zum Nutzen der ECMO bei schweren COVID-19-Fällen sind inkonsistent. Initiale Daten aus dem Frühjahr 2020 deuteten auf ein eher schlechteres Outcome aufgrund einer unkontrollierten Hyperinflammation hin [50]. Neuere Daten zeigen eher positive Effekte auf das Überleben, möglicherweise bedingt durch die Kombinationstherapie mit antiinflammatorischen Substanzen [51].

Leider ist auch nach Abklingen der SARS-CoV-2-Infektion das Erkrankungsgeschehen noch nicht beendet, denn Langzeiteffekte einer SARS-CoV-2-Infektion sind facettenreich, schwierig vorherzusagen und noch schwieriger zu behandeln. Fallberichte von Patienten, die nach der akuten Infektion über persistierende Belastungsbeschwerden berichten, sind tägliche Routine geworden. Diese Phase wird heute als Post-COVID-19-Syndrom bezeichnet und scheint nicht nur nach schweren, sondern auch nach leicht verlaufenden Infektionen aufzutreten. Betroffene Patienten berichten über Abgeschlagenheit und Leistungsschwäche, Konzentrationsstörungen, Schlafstörungen, fortbestehenden Geruchs- oder Geschmacksstörungen, gastrointestinale Beschwerden oder andere Symptome. Die Diagnostik und die Behandlung dieses Syndroms bedürfen eines spezialisierten interdisziplinären Teams.

Aus kardiologischer Sicht zeigten erste Bildgebungsanalysen, dass COVID-19-Patienten auch Monate nach der Erkrankung Myokardschäden aufwiesen, die unabhängig von der Schwere des Erkrankungsverlaufs waren. Interaktionen und Prävalenzen für persistierende Beschwerden bei Patienten mit kardiovaskulären Komorbiditäten stehen noch aus. Dementsprechend stehen auch noch keine Therapieempfehlungen zur Verfügung. Trotzdem sollten Patienten, die etwa 2 bis 3 Wochen nach diagnostizierter COVID-19 noch keine deutliche Besserung aufweisen, eine weiterführende Abklärung erhalten, um u. a. kardiovaskuläre Komplikationen, wie eine chronische Perimyokarditis oder eine inflammatorische Kardiomyopathie, frühzeitig ausschließen zu können. Aktuelle Empfehlungen zur Diagnostik und Behandlung von Patienten mit SARS-CoV-2/COVID-19 stehen auf den Internetseiten des Robert Koch-Instituts sowie medizinischer Fachgesellschaften und sollten bei der Behandlung von Patienten mit COVID-19 berücksichtigt werden.
